# Vibration Identification of Folded-MEMS Comb Drive Resonators

**DOI:** 10.3390/mi9080381

**Published:** 2018-08-01

**Authors:** Jianxin Han, Lei Li, Gang Jin, Jingjing Feng, Baizhou Li, Haili Jia, Wenkui Ma

**Affiliations:** 1 Tianjin Key Laboratory of High Speed Cutting and Precision Machining, School of Mechanical Engineering, Tianjin University of Technology and Education, Tianjin 300222, China; hanjianxin@tju.edu.cn (J.H.); lbzy_719@sina.com (B.L.); hljia@tute.edu.cn (H.J.); 2 School of Transportation and Vehicle Engineering, Shandong University of Technology, Zibo 255049, China; lileizi888@tju.edu.cn; 3 Tianjin Key Laboratory for Advanced Mechatronic System Design and Intelligent Control, School of Mechanical Engineering, Tianjin University of Technology, Tianjin 300384, China; jjfeng@tju.edu.cn; 4 Department of Mechanical Engineering, Henan Mechanical and Electrical Vocational College, Zhengzhou 451191, China; mawenkui1986@163.com

**Keywords:** MEMS, nonlinear vibration, comb drive, primary resonance

## Abstract

Natural frequency and frequency response are two important indicators for the performances of resonant microelectromechanical systems (MEMS) devices. This paper analytically and numerically investigates the vibration identification of the primary resonance of one type of folded-MEMS comb drive resonator. The governing equation of motion, considering structure and electrostatic nonlinearities, is firstly introduced. To overcome the shortcoming of frequency assumption in the literature, an improved theoretical solution procedure combined with the method of multiple scales and the homotopy concept is applied for primary resonance solutions in which frequency shift due to DC voltage is thoroughly considered. Through theoretical predictions and numerical results via the finite difference method and fourth-order Runge-Kutta simulation, we find that the primary frequency response actually includes low and high-energy branches when AC excitation is small enough. As AC excitation increases to a certain value, both branches intersect with each other. Then, based on the variation properties of frequency response branches, hardening and softening bending, and the ideal estimation of dynamic pull-in instability, a zoning diagram depicting extreme vibration amplitude versus DC voltage is then obtained that separates the dynamic response into five regions. Excellent agreements between the theoretical predictions and simulation results illustrate the effectiveness of the analyses.

## 1. Introduction

The resonant mode operation of microelectromechanical systems (MEMS) has found many applications in recent decades due to its advantages, such as a high signal-to-noise ratio, high quality factor, high sensitivity, and high dynamic stability [1,2,3,4,5]. To realize different functions of resonant MEMS, micro-components are designed into various configurations, in which comb drive with folded suspension configuration gradually becomes one of the most popular micro-components in the sensing and actuation field due to its capability in suppressing nonlinearities and increasing actuation force. Unfortunately, nonlinearities induced by structure restoring force and electrostatic force still can be observed as the increase of the displacement of micro-components. For the large-stroke demands of MEMS devices, these nonlinearities cannot be ignored anymore, and sometimes, they may even have significant effects on system performances, inducing some particular phenomena such as pull-in instability, hardening behavior, softening behavior, or mixed behavior [6,7].

Until now, the effects of nonlinearities on the static and dynamic performances of MEMS devices have been discussed a much of the literature. Some of them contribute to suppressing nonlinearities [8,9], while some prefer to investigate the advantages of nonlinearities in the design of new MEMS devices [10,11,12]. For instance, Shmulevich and Elata [8] designed a new dynamically balanced folded beam suspension to suppress nonlinearity in the resonator and finally experimentally demonstrated the viability of new design. Hassanpour et al. [9] addressed primary and secondary internal resonance analyses of a beam-type resonant structure due to the stretching of the beam, and investigated the tension effect on the reduction of nonlinear vibration. Wang et al. [10] numerically and experimentally investigated the chaos and parameter estimation of a tunable oscillator, and finally discussed its potential applications in secure communication. Zhang et al. [11] gave a thorough investigation on the design of a highly sensitive mass sensor based on the parametric resonance analysis of a comb drive resonator governed by a nonlinear Mathieu equation. Rhoads et al. [12] indicated that the nonlinear parametric resonance of a bandpass filter of comb drive MEMS oscillators could effectively improve some filtering characteristics of the oscillator.

To design and optimize these MEMS devices, one should firstly understand in detail the statics/dynamics that may exist in the system [13,14,15,16,17,18,19]. Then, combined with the mechanical principles previously derived and the real demands of MEMS communities, one can summarize some design rules that may finally offer help in reducing the production period and cost for new MEMS devices. Among these studies, some of them placed an emphasis on theoretical investigations. Numerical or experimental results are used more for verifying theoretical conclusions. Guided by the theoretical results, one can not only realize some traditional uses of such MEMS devices, but also explore more valuable applications, extending the application bound. For example, Zhong et al. [20] studied the effect of the inclination of fingers on the natural frequency and stiffness of a comb drive resonator. The results showed that the inclination can strengthen system stability and avoid pull-in instability, which provides some design thoughts for such MEMS devices. Guo et al. [21] theoretically designed a quadratic-shaped finger comb parametric resonator. Through fabrication process and experimental studies, they discussed depth this device in and illustrated its potential uses in chemical sensor, strain gauge, and gyroscope applications. The above studies indubitably indicate the importance of theoretical analysis.

Theoretical investigation should gain an insight into the object of study, from either a qualitative or a quantitative viewpoint. This is a very important step for the design and optimization of MEMS devices. Recently, taken both transverse and longitudinal capacitances and the nonlinearity of folded suspension beams into consideration, Elshurafa et al. [7] gave an excellent primary resonance investigation on one type of folded-MEMS comb drive resonators via the method of multiple scales (MMS) and Matlab/Simulink simulations. Some interesting phenomena such as bending and jumping behaviors were discussed in detail, which shed light on the possibility of theoretical analyzing this MEMS resonator. However, during the application of MMS, they assumed that the nondimensional excitation (angular) frequency was close to one, indicating that the nondimensional natural (angular) frequency of the resonator was equal to one. Based on previous studies in the literature, one can notice that DC voltage can effectively tune the natural frequency of the resonator; sometimes, it can even induce obvious dynamic changes [22,23]. Theoretically, the nondimensional natural (angular) frequency in a prebuckling case (a required working pattern in folded-MEMS comb drive resonators) can be set from one to zero as the DC voltage increases [24]. Although a relatively small DC voltage (far away from pull-in voltage) can yield consistent trends for their solutions compared with experiment results, when DC voltage is big enough, the theoretical results in Reference [7] may induce low prediction accuracy, which implies some of the limitations of their conclusions. What is more, based on our previous studies on a symmetric MEMS device, of which the governing equation of motion is very similar to this comb drive resonator, we successfully observed two primary frequency branches that existed in the system [25]. Perhaps, this dynamic MEMS device can also possess this property, which can be rationally utilized in enlarging the bandwidth and energy output for sensing and energy harvesting [25]. In summary, all we want is to try to figure out two key problems of this folded-MEMS comb drive resonator. The first is to accurately predict the variation of natural (angular) frequency as the DC voltage increases (between zero and pull-in voltage). The other is to gain an insight into the primary resonance of the system—one of the most important working patterns for dynamic MEMS devices—discuss the primary frequency response, and finally conclude with some valuable tips for the subsequent design and parameter optimization of such systems.

The structure of this paper is as follows. In Section 2, the governing equation of motion of a folded-MEMS comb drive resonator is introduced. In Section 3, the MMS combined with the homotopy concept is applied to deduce the primary resonance solution [25,26]. A case study is carried out to reveal the existence of low and high-energy branches and intersection properties under primary resonance conditions. In Section 4, vibration identification is investigated in detail based on theoretical solutions. A zoning diagram to distinguish frequency response bending and intersection phenomena is drawn and examined in depth. Finally, discussions and conclusions are presented in Section 5.

## 2. Equation of Motion

**Figure 1:**
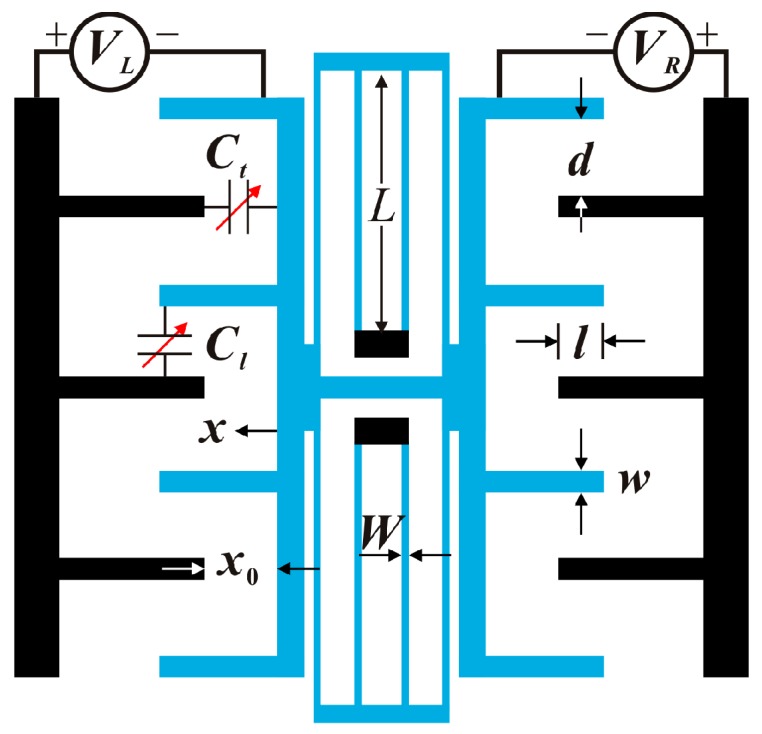
Schematic diagram of a folded-microelectromechanical systems (MEMS) comb drive resonator [7].

As shown in Figure 1, a folded-MEMS comb drive resonator composes of a suspended moving mass and two symmetric fixed electrodes [7]. l is the finger overlap distance. $x_{0}$ is the initial lateral spacing between the fixed and moving combs. d is the finger gap spacing. h is the structure layer thickness. w is the finger width. W and L are the beam width and length, respectively. $C_{t}$ and $C_{l}$ are the transverse and longitudinal capacitances between the fixed and moving parts of the structure, respectively. The middle moving part is actuated by left and right voltages $V_{L}=V_{D}+V_{A}\cos(\Omega t)$ and $V_{R}=V_{D}-V_{A}\cos(\Omega t)$, in which $V_{D}$ is the DC voltage, and $V_{A}$ and Ω are the amplitude and frequency of the AC voltage. The governing equation of motion of this dynamic system can be given by [7]:

(1) $$m\frac{d^{2}x}{dt^{2}}+c\frac{dx}{dt}+k_{1}x+k_{3}x^{3}=\left[\alpha_{2}+\frac{\alpha_{1}}{{\left(x_{0}-x\right)}^{2}}\right]{\left[V_{D}+V_{A}\cos(\Omega t)\right]}^{2}-\left[\alpha_{2}+\frac{\alpha_{1}}{{\left(x_{0}+x\right)}^{2}}\right]{\left[V_{D}-V_{A}\cos(\Omega t)\right]}^{2}\;$$

in which

$$k_{1}=\frac{24EI}{L^{3}},\;k_{3}=\frac{216EI}{35L^{5}},\;\alpha_{1}=\frac{N\varepsilon_{0}A_{t}}{2},\;\;\alpha_{2}=\frac{\varepsilon_{0}(N-1)}{2}\left\{\frac{h}{d}+\frac{1}{\pi }\ln\left[\left[{\left(\frac{w}{d}+1\right)}^{2}-1\right]{\left[\frac{2d}{w}+1\right]}^{\left(1+\frac{w}{d}\right)}\right]\right\}$$

where t is the time. x is the transverse displacement of the comb drive. m, c, $k_{1}$ and $k_{3}$ are the effective lumped mass, damping, linear and cubic stiffness, respectively. $\varepsilon_{0}$ is the dielectric constant. N is the total number of fingers.

For convenience, we introduce the following nondimensional variables:

(2) $$\xi =\frac{x}{x_{0}},\;\tau =\omega_{0}t$$

where time scale $\omega_{0}=\sqrt{k_{1}/m}$ is the natural (angular) frequency of the comb drive resonator without DC voltage.

Substituting Equation (2) into Equation (1) yields:

(3) $$\begin{array}{cc} \ddot{\xi }+2\mu \dot{\xi }+\xi +\alpha \xi^{3} & =\gamma \left[\frac{1}{{\left(1-\xi \right)}^{2}}-\frac{1}{{\left(1+\xi \right)}^{2}}\right]+4\gamma \delta \rho \cos(\omega \tau )+2\gamma \rho \left[\frac{1}{{\left(1-\xi \right)}^{2}}+\frac{1}{{\left(1+\xi \right)}^{2}}\right]\cos(\omega \tau )\\ & +\gamma \rho^{2}\left[\frac{1}{{\left(1-\xi \right)}^{2}}-\frac{1}{{\left(1+\xi \right)}^{2}}\right]\cos^{2}(\omega \tau ) \end{array}\;$$

where (·) represents the derivative with respect to nondimensional time *τ*, and $\mu =\frac{c}{2\sqrt{k_{1}m}}$, $\alpha =\frac{9x_{0}^{2}}{35L^{2}}$, $\gamma =\frac{N\varepsilon_{0}wL^{3}V_{D}^{2}}{4EW^{3}x_{0}^{3}}$, $\delta =\frac{\alpha_{n2}}{\alpha_{n1}}$, $\alpha_{n1}=\frac{\alpha_{1}}{k_{1}x_{0}^{3}}$, $\alpha_{n2}=\frac{\alpha_{2}}{k_{1}x_{0}}$, $\omega =\frac{\Omega }{\omega_{0}}$, $\rho =\frac{V_{A}}{V_{D}}$.

The normal operation mode of this comb drive resonator should be the prebuckling pattern [7], which means that zero equilibrium is the only stable static position of the resonator. Then, the natural (angular) frequency of the resonator with DC voltage can be given by [24]:

(4) $$\omega_{n}=\sqrt{1-4\gamma }$$

Note that Equation (3) is an exact governing equation of motion without any simplification. Thus, numerical simulation based on this equation can be used to verify the effectiveness and correctness of the theoretical results.

## 3. Primary Frequency Response

The electrostatic terms in Equation (3) increase the difficulties of theoretical derivation. To obtain concise while accurate theoretical solution, we must firstly neglect some terms in a reasonable way. It is well known that generally, AC voltage $V_{A}$ is much smaller than DC voltage $V_{D}$. Then, we can obtain a simplified version of Equation (3) as follows [7]:

(5) $$\ddot{\xi }+2\mu \dot{\xi }+\xi +\alpha \xi^{3}=\gamma \left[\frac{1}{{\left(1-\xi \right)}^{2}}-\frac{1}{{\left(1+\xi \right)}^{2}}\right]+4\gamma \delta \rho \cos(\omega \tau )\;$$

The above equation is still complex as the existence of an electrostatic term induced by nondimensional DC voltage γ. Perhaps, the normal way to figure out this problem is to apply Taylor expansion [27] or multiply Equation (5) by ${\left(1-\xi^{2}\right)}^{2}$ [28]. However, the former simplification may induce some obvious mistakes during theoretical prediction [29]. The later multiplication definitely increases the complexity of the governing equation. To balance the accuracy and simplicity of the theoretical solution, we successfully figured out an equation of motion similar to the above by the application of the MMS combined with the homotopy concept [25]. The residue theorem was used to find the secular terms in the system. Here, we try to solve the above equation based on our previous work.

### 3.1. Solution Procedure

A solution procedure with traditional MMS usually needs to introduce a detuning parameter to describe the nearness of the excitation frequency to the natural (angular) frequency of the system. In Reference [7], the approximation of the excitation frequency ω is given by:

(6) ω=1+εσ 

where ε is just a scaling parameter without any true value. σ is a detuning parameter with a relatively small value compared with one.

Then, the equation of motion with scaling *ε* can be given by [7]:

(7) $$\ddot{\xi }+\xi =\varepsilon \left\{-2\mu \dot{\xi }-\alpha \xi^{3}+\gamma \left[\frac{1}{{\left(1-\xi \right)}^{2}}-\frac{1}{{\left(1+\xi \right)}^{2}}\right]+4\gamma \delta \rho \cos(\omega \tau )\right\}\;$$

By the application of MMS, one can derive the average equation and primary frequency response as described in Reference [7]. For convenience and conciseness, we give the corresponding theoretical solutions of Reference [7] in the Appendix A.

Obviously, the above approximations assume that the natural (angular) frequency of this resonator with DC voltage is equal to one. When the DC voltage is relatively small and far away from the pull-in voltage, the above assumption is reasonable. However, when the DC voltage is big enough, the natural (angular) frequency $\omega_{n}$ is far away from one. Then, Equation (6) is not available any more. To illustrate this, we obtain the natural (angular) frequency Equation (A4) through the backbone equation of Equation (A3), which is different from the right one in Equation (4). Only when DC voltage γ is small enough are they both approximate to one. Once γ is away from zero, their difference becomes obvious.

To obtain a more accurate primary frequency response that is suitable for both small and large DC voltage, here we apply the MMS combined with the homotopy concept. It is worth mentioning that the homotopy method does not need to describe the nearness of excitation frequency to natural frequency. It regards that the final vibration frequency is equal to the excitation frequency [26]. By introducing an embedding parameter, this method can effectively describe the transition process between an ideal linear system and a practical nonlinear system. Following this procedure, we can obtain a new form of the governing equation based on Equation (5) as follows:

(8) $$\ddot{\xi }+\omega^{2}\xi =p\left\{\left(\omega^{2}-1)\xi -2\mu \dot{\xi }-\alpha \xi^{3}+\gamma \left[\frac{1}{{\left(1-\xi \right)}^{2}}-\frac{1}{{\left(1+\xi \right)}^{2}}\right]+4\gamma \delta \rho \cos(\omega \tau \right)\right\}\;$$

where p can be regarded as an embedding parameter, p=0 can yield a linearized system, and p=1 can yield a nonlinear system. By the application of the MMS and the homotopy concept, we can finally derive the average equation as follows:

(9) $$\{\begin{array}{c} \frac{dA}{dT_{1}}=-\mu A-\frac{2\gamma \delta \rho }{\omega }\sin\beta \\ \frac{d\beta }{dT_{1}}=\frac{3\alpha A^{2}}{8\omega }+\frac{1-\omega^{2}}{2\omega }-\frac{2\gamma }{\omega {\left(1-A^{2}\right)}^{3/2}}-\frac{2\gamma \delta \rho }{\omega A}\cos\beta \end{array}\;$$

where $T_{1}=p\tau$, $A(T_{1})$ is the vibration amplitude and $\beta (T_{1})$ is the vibration phase.

The primary frequency response can be given by:

(10) $$\frac{3\alpha A^{2}}{8\omega }+\frac{1-\omega^{2}}{2\omega }-\frac{2\gamma }{\omega {\left(1-A^{2}\right)}^{3/2}}\pm \sqrt{{\left(\frac{2\gamma \delta \rho }{\omega A}\right)}^{2}-\mu^{2}}=0\;$$

The backbone curve can be expressed as:

(11) $$\omega_{0}=\sqrt{1+\frac{3\alpha A^{2}}{4}-\frac{4\gamma }{{\left(1-A^{2}\right)}^{3/2}}}\;$$

Similarly, we can also derive the natural (angular) frequency through the backbone equation by letting A=0 on Equation (11), yielding this frequency $\omega_{0,0}$ as follows:

(12) $$\omega_{0,0}=\sqrt{1-4\gamma }\;$$

Obviously, Equation (12) is the same as Equation (4). Based on nonlinear vibration theory [30], one can notice that our backbone equation is correct.

Besides, the dynamic stability of the system can be identified by the following Jacobi matrix:

(13) $$J=\left[\begin{array}{cc} -\mu & -\frac{2\gamma \delta \rho }{\omega }\cos\beta \\ \frac{3\alpha A}{4\omega }-\frac{6\gamma A}{\omega {\left(1-A^{2}\right)}^{5/2}}+\frac{2\gamma \delta \rho }{\omega A^{2}}\cos\beta & \frac{2\gamma \delta \rho }{\omega A}\sin\beta \end{array}\right]\;$$

Finally, the eigenvalue λ of the above Jacobi matrix can be given by:

(14) $$\lambda^{2}+R\lambda +S=0\;$$

where:

(15) $$R=2\mu ,\;S=\mu^{2}+\left[\frac{3\alpha A^{2}}{8\omega }+\frac{1-\omega^{2}}{2\omega }-\frac{2\gamma }{{\left(1-A^{2}\right)}^{3/2}}\right]\cdot \left[\frac{9\alpha A^{2}}{8\omega }+\frac{1-\omega^{2}}{2\omega }-\frac{2\gamma (1+2A^{2})}{{\left(1-A^{2}\right)}^{5/2}}\right]$$

when R and S are both positive, the vibration of the system is stable.

### 3.2. Theoretical Results

To grasp the dynamic properties of the system, we should consider both the characteristics of the corresponding Hamilton system and the actual dynamic system. Following up on our previous work, we give the following analyses in detail.

Dynamic pull-in instability is the first consideration for resonant MEMS devices. Theoretically, vibration should be within the area surrounded by heteroclinic orbits. Otherwise, dynamic pull-in is triggered [24]. Therefore, the theoretical maximum vibration amplitude—in other words, dynamic pull-in amplitude—can be defined according to the distance between the non-zero unstable equilibrium and the zero stable equilibrium of the system. Obviously, dynamic pull-in vibration amplitude $A_{p}$ can be estimated as [24,25]:

(16) $$A_{p}=\sqrt{\varpi^{2}\cdot \sqrt[{3}]{-\frac{q_{0}}{2}+\sqrt{{\left(\frac{q_{0}}{2}\right)}^{2}+{\left(\frac{p_{0}}{3}\right)}^{3}}}+\varpi \cdot \sqrt[{3}]{-\frac{q_{0}}{2}-\sqrt{{\left(\frac{q_{0}}{2}\right)}^{2}+{\left(\frac{p_{0}}{3}\right)}^{3}}}-\frac{1-2\alpha }{3\alpha }}\;$$

where $p_{0}=-\frac{{\left(1+\alpha \right)}^{2}}{3\alpha^{2}}$, $q_{0}=\frac{2[{\left(1+\alpha \right)}^{3}-54\alpha^{2}\gamma ]}{27\alpha^{3}}$, $\varpi =\frac{-1+\sqrt{3}i}{2}$, $i=\sqrt{-1}$.

Away from dynamic pull-in instability, we can now discuss the real vibration amplitude of the system. Based on nonlinear vibration theory regarding primary frequency response [30], the extreme point (or peak value) on a frequency response curve must be on the backbone curve. Thus, the determination of the extreme point can be realized through analyzing Equations (10) and (11). Obviously, there are three aspects to consider:

- The number of extreme points in the system and its/their evolution properties;
- The bending property of the backbone curve, such as hardening, softening, or mixed vibration;
- The specific location of the extreme point, which can total reflect the vibration type of the resonator.

For a specific resonator and a given DC voltage and AC excitation amplitude, based on Equations (10) and (11), we can numerically determine the extreme frequency *ω_e_* and amplitude *A_e_* by using the following equations:

(17) $$\omega_{e}=\sqrt{1+\frac{3\alpha A_{e}^{2}}{4}-\frac{4\gamma }{{\left(1-A_{e}^{2}\right)}^{3/2}}}\;$$

(18) $$1+\frac{3\alpha A_{e}^{2}}{4}-{\left(\frac{2\gamma \delta \rho }{\mu A_{e}}\right)}^{2}-\frac{4\gamma }{{\left(1-A_{e}^{2}\right)}^{3/2}}=0\;$$

**Figure 2:**
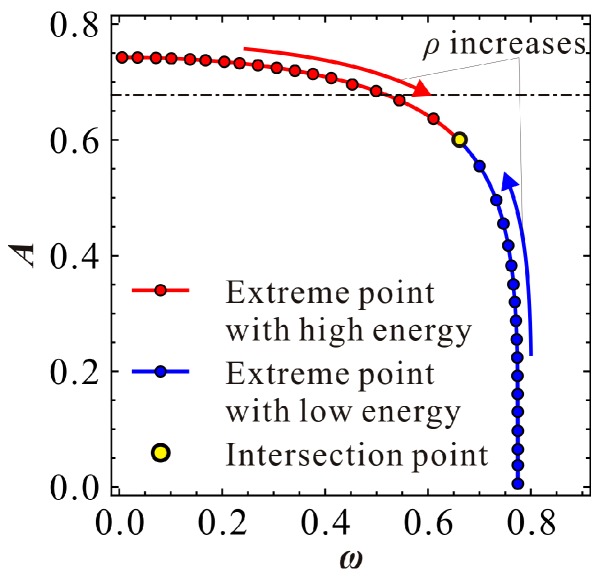
Variations of the extreme point as the increase of nondimensional AC excitation amplitude ρ for α=0.8, γ=0.1, μ=0.005 and δ=100.

Through numerical calculation of the above equations, we can investigate the variation properties of the extreme amplitude. A general understanding can be grasped in Figure 2. Note that the nondimensional parameters are selected after considering the physical parameters in Reference [7]. Similar to our previous work [25], we find that this resonator contain two types of extreme points when AC excitation amplitude ρ is small enough. One type has relative high energy, while the other has low energy. When ρ increases into a critical value, two extreme points degenerate into one point, which is named as the intersection point. A further increase of ρ can induce the missing of the extreme point. The above phenomena indicate the existence of two primary frequency branches in the system. Here, we are more concerned with the critical condition. That is, when ρ increases to a critical value, two extreme points degenerate into one point. Obviously, finding this intersection point is important for frequency response identification.

When two extreme points degenerate into one point, a critical condition with $d\rho /dA_{e}=0$ can be found by using Equation (18). Finally, the intersection AC excitation amplitude $\rho_{i}$ can be derived as:

(19) $$\rho_{i}=\sqrt{\frac{3A_{i}^{4}\mu^{2}}{16\gamma^{2}\delta^{2}}\left[\frac{8\gamma }{{\left(1-A_{i}^{2}\right)}^{5/2}}-\alpha \right]}\;$$

where $A_{i}$ represents the intersection amplitude, a special extreme amplitude $A_{e}$. Substituting Equation (19) into Equation (18), one can numerically derive the intersection amplitude $A_{i}$. By using Equation (17), one can finally obtain the intersection frequency $\omega_{i}$.

**Figure 3:**
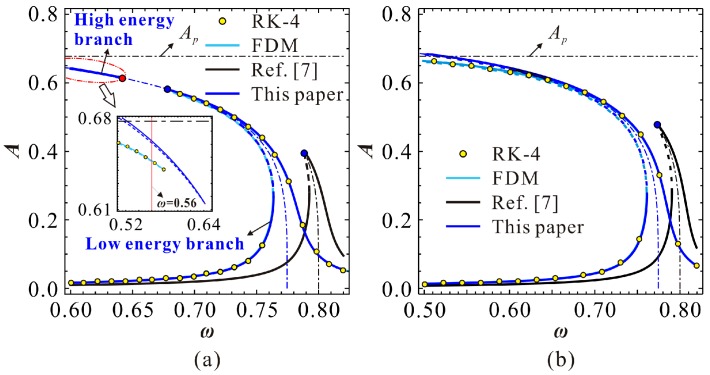
Frequency response curve for α=0.8, γ=0.1, μ=0.005 and δ=100 (**a**) $\rho =9.86\times 10^{-5}$; (**b**) $\rho =1.20\times 10^{-4}$ (note that $\rho_{i}=9.89\times 10^{-5}$; solid line: stable solutions; dashed line: unstable solutions).

To verify the correctness of our theoretical results, we numerically simulate Equation (3) via both the finite difference method (FDM) and fourth-order Runge–Kutta (RK-4) simulations. The final results are shown in Figure 3. When $\rho <\rho_{i}$, as shown in Figure 3a, there are two extreme points in the system. Meanwhile, the primary frequency response contains two branches, one with low energy, while the other has high energy. If $\rho >\rho_{i}$, two branches intersect with each other, just like Figure 3b. From this figure, one can notice that our prediction is correct.

**Figure 4:**
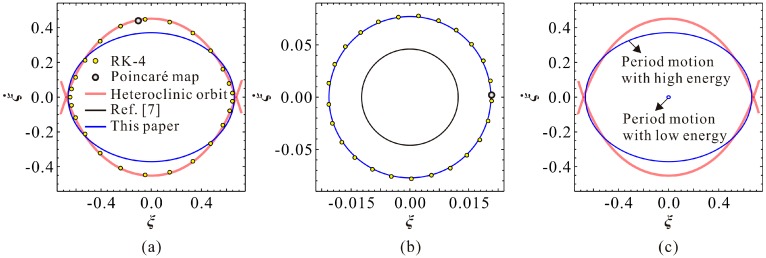
Phase diagram corresponding to Figure 3a for ω=0.56. (**a**) Phase diagram with relatively high vibration energy, initial condition (−0.100427, 0.441685); (**b**) phase diagram with relatively low vibration energy, initial condition (0, 0); (**c**) a specific comparison of the phase diagrams in (**a**,**b**).

Actually, the primary resonance branches in Figure 3a are classified based on system vibration energy, which can be thoroughly grasped via a stable phase diagram. Taking ω=0.56 in Figure 3a as an example, we can finally obtain the phase diagram of the system, as shown in Figure 4. From this figure, one can notice that: (i) the vibrations on two branches are both period-1 motion, as the Poincaré map has only one mapping point; (ii) periodic motions on two branches have different vibration energy, one with relatively high energy, and the other with relatively low energy; (iii) our present result can predict the existence of both high and low-energy motion; (iv) low energy motion shows excellent agreement with the RK-4 results, although high-energy motion can exhibit some errors; (v) the theoretical result in Reference [7] can only predict the existence of low-energy motion, and with some prediction error.

Here, it should be announced that the period motion on the high-energy branch is strictly dependent on the initial condition of the system. In order to obtain this high-energy vibration, one can investigate its basin of attraction in depth via the cell mapping method. However, this is beyond the scope of our present research. We are more concerned with the intersection phenomenon of high and low-energy frequency response branches, which can effectively increase the bandwidth through the frequency sweep-down operation, providing some potential use in areas such as MEMS filtering or energy harvesting.

The bending way of the frequency response curve can be determined by the sign of the derivative of $\omega_{0}$ to A. A switch amplitude $A_{s}$ can be derived by using $d\omega_{0}/dA=0$, which yields:

(20) $$A_{s}=\sqrt{1-{\left(\frac{8\gamma }{\alpha }\right)}^{2/5}}$$

note that $A_{s}$ is also a special extreme amplitude on the backbone curve. Substituting Equation (20) into Equations (10) and (11), one can also obtain the corresponding switch AC excitation frequency $\omega_{s}$ and amplitude $\rho_{s}$ as follows:

(21) $$\omega_{s}=\sqrt{1+\frac{3\alpha A_{s}^{2}}{4}-\frac{4\gamma }{{\left(1-A_{s}^{2}\right)}^{3/2}}}\;$$

(22) $$\rho_{s}=\sqrt{\left[1+\frac{3\alpha A_{s}^{2}}{4}-\frac{4\gamma }{{\left(1-A_{s}^{2}\right)}^{3/2}}\right]{\left(\frac{\mu A_{s}}{2\gamma \delta }\right)}^{2}}\;$$

Switch amplitude *A_s_* actually describes the existence of the bending of the primary frequency response. If this amplitude exists, i.e., α≥8γ, then the system can appear to have hardening vibration and hardening-to-softening or softening-to-hardening vibration. If no real solution can be derived from Equation (20), i.e., α<8γ, then the system can only exhibit softening vibration. Undoubtedly, the existence of bending vibration still relies on the relationship between $A_{e}$ and $A_{s}$.

For a specific MEMS resonator and a given DC voltage, one can obtain a specific $A_{s}$ and $A_{i}$. The relationship of the above critical amplitudes can be used to identify the vibration type of the MEMS resonator. To find a critical condition, we firstly assume $A_{s}=A_{i}$. Substituting Equations (19) and (20) into Equation (18), one can obtain a critical DC voltage $\gamma_{is}$ as follows:

(23) $$\gamma_{is}=\frac{{\left(4+3\alpha \right)}^{2}}{1000\alpha }\sqrt{15+\frac{20}{\alpha }}\;$$

**Figure 5:**
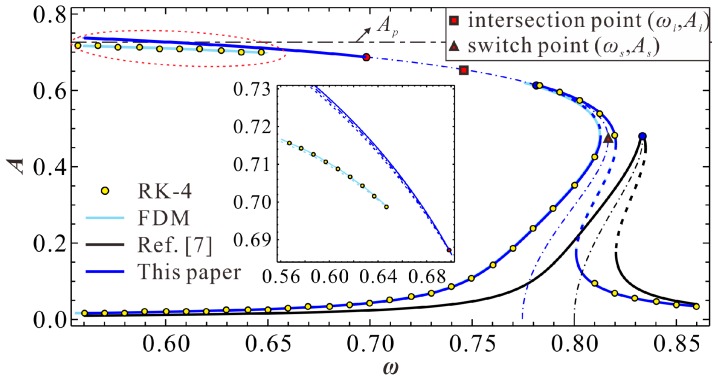
Bending motion of the primary frequency response for α=1.5, γ=0.1, μ=0.005, δ=100 and $\rho =9\times 10^{-5}$ (note that $\rho_{i}=1.22\times 10^{-4}$ and $\rho_{s}=9.62\times 10^{-5}$; solid line: stable solutions; dashed line: unstable solutions).

We can easily obtain the minimum value $\min(\gamma_{is})=0.25$ when cubic stiffness α=2. For this comb drive resonator, the prebuckling operation mode needs DC voltage γ to be smaller than 0.25. Besides, based on the expression of nondimensional parameter α and the physical parameters in Reference [7], one can notice that in general condition, structure nonlinearity of the resonator is totally small, yielding 0<α<2. Thus, we cannot find a critical DC voltage $\gamma_{is}$ to let $A_{s}=A_{i}$. In other words, $A_{s}$ curve and $A_{i}$ curve versus DC voltage γ cannot intersect with each other. Note that when γ=α/8<0.25, $A_{s}=0$ and $A_{i}>0$. Thus, for a specific DC voltage γ, $A_{i}>A_{s}$. Therefore, if bending motion exists, it must be trigged prior to intersection phenomenon. This also implies that only the low energy frequency response branch can appear bending motion. This conclusion is totally different from our previous results on a prebuckling microbeam-based resonator [25]. As shown in Figure 5, the bending types of the frequency branches with AC excitation $\rho_{s}<\rho <\rho_{i}$ verify our theoretical prediction. By contrast, the theoretical results in Reference [7] can only predict the existence of the low-energy branch, while the result exhibits a relatively low prediction accuracy.

The above analysis is only a local case study. Vibration identification of this MEMS resonator under different parameter combinations is crucial to the dynamic investigation and parameter optimization of the system. Therefore, the following analysis is based on all of the above theoretical results and mainly focuses on vibration identification under primary resonance conditions.

## 4. Vibration Identification

If we take care of the frequency response branch with high energy, we can notice that the theoretical results will lose their prediction accuracy when the vibration amplitude is close to dynamic pull-in amplitude $A_{p}$. This is a weakness of the present method, which may result from the time averaging process when using MMS with a periodic assumption for theoretical solution [25]. Fortunately, we can accurately predict the intersection phenomenon of the high and low-energy frequency response branches. As the high-energy branch is difficult to manifest in practice, we focus on the intersection behavior of frequency response branches. Besides, as the low-energy branch and its variation property are both crucial for the performance of the resonator, we investigate this branch in detail, especially on the bending and jumping motion.

### 4.1. Nondimensional Analysis

**Figure 6:**
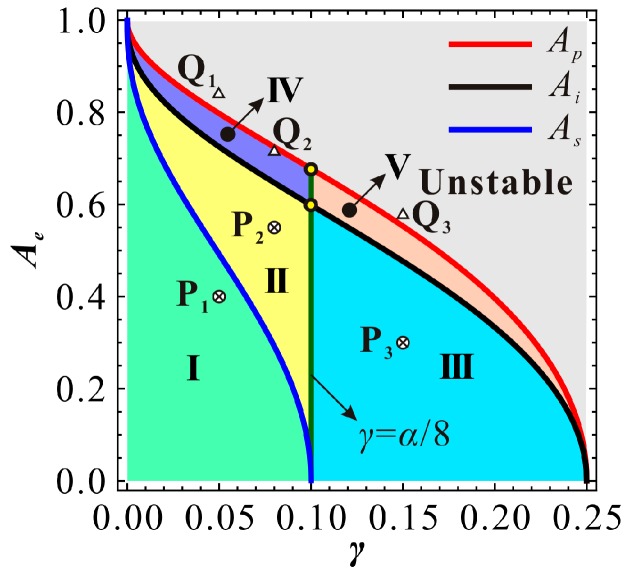
A nondimensional zoning diagram depicting the extreme amplitude versus nondimensional DC voltage for α=0.8, μ=0.005 and δ=100 (P_1_, P_2_, and P_3_ correspond to extreme amplitudes with low energy. The corresponding Q_1_, Q_2_, and Q_3_ have high-energy extreme amplitudes).

As DC voltage is used to tune the resonance frequency of the resonator, it can be theoretically set to any value between zero and the static pull-in voltage. Considering the ideal dynamic pull-in amplitude $A_{p}$, switch amplitude $A_{s}$, intersection amplitude $A_{i}$, switch condition α=8γ and static pull-in condition γ=0.25, one can finally obtain a zoning diagram (Figure 6) depicting extreme amplitude $A_{e}$ versus DC voltage γ. A point below $A_{i}$ curve corresponds to an extreme amplitude $A_{e}$ with low energy. Meanwhile, corresponding high energy extreme amplitude can also be found above $A_{i}$ curve. For instance, for a given DC voltage γ, P_1_ has low-energy extreme amplitude. Through calculation with Equation (18), we can also obtain an extreme point Q_1_ with high energy. As the increase of AC excitation amplitude ρ, both the low and high extreme amplitudes are close to each other along the backbone curve (seeing Figure 2). When $\rho =\rho_{i}$, two points degenerate into one point, which is on the $A_{i}$ curve. Then, if we continue to increase ρ, no extreme amplitude can be found any more; the low and high-energy branches intersect with each other. Through analyzing the minimum or maximum of extreme amplitude, we can finally describe the vibration property of the system in detail. Considering the switch condition and intersection condition, it is clear that in Region-I, the low-energy frequency response branch is local hardening, and the high-energy branch is softening. In Region-II, the low-energy frequency response branch exhibits a hardening-to-softening bending motion, and the high-energy branch is softening. In Region-III, the low-energy frequency response branch exhibits an overall softening vibration, and the high-energy branch exhibits a softening vibration. In Region-IV, the low and high-energy branches intersect with each other. No extreme point can be found in the system. The frequency response exhibits an overall hardening-to-softening bending motion. In Region-V, the low and high-energy branches intersect with each other as well. The frequency response exhibits an overall softening bending motion. α=8γ is a criteria to estimate the approximate linear vibration with small vibration assumption [24]. It should be announced that without intersection phenonmon, the high-energy branch is softening all of the time. No bending transition of this branch can be observed in the system.

**Figure 7:**
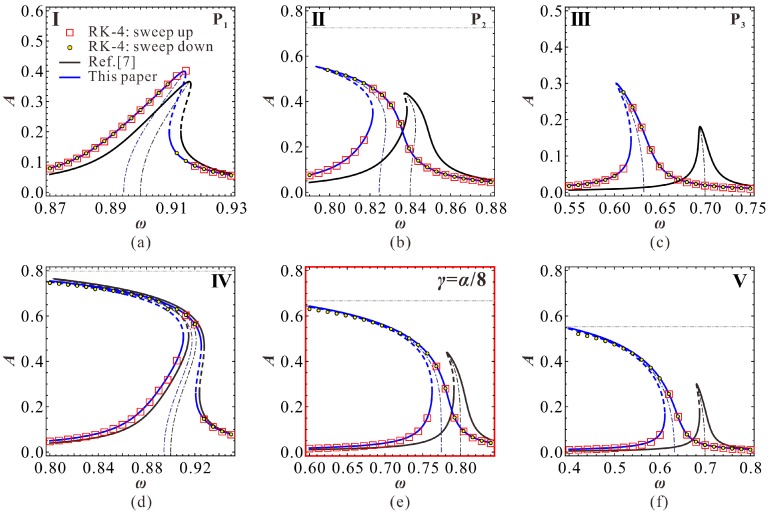
Frequency response curve with low energy corresponding to Figure 5 (**a**) γ=0.05, $\rho =1.83\times 10^{-4}$; (**b**) γ=0.08, $\rho =1.37\times 10^{-4}$; (**c**) γ=0.15, $\rho =3.01\times 10^{-5}$; (**d**) γ=0.05, $\rho =4\times 10^{-4}$; (**e**) γ=0.1, $\rho =1.1\times 10^{-4}$; (**f**) γ=0.15, $\rho =5\times 10^{-5}$ (solid line: stable solutions; dashed line: unstable solutions).

To make our theoretical results more convincing, RK-4 simulations are used to verify our theoretical solutions. As the jumping motion can be potentially used in MEMS switch applications [3], we numerically simulate through frequency up and down-sweep procedures. We pay more attention to the low-energy primary frequency branch. The only focus on the high-energy frequency response is the intersection phenomenon. The corresponding simulation results are shown in Figure 7, which verifies the correctness of our theoretical predictions. Actually, no ideal linear vibration can be found in the system. As the AC excitation amplitude ρ increases, softening vibration becomes obvious in the system. Here, we only give a case study to observe this vibration, as shown in Figure 7e. We do not exhibit the high-energy branch any more. If one has interest in observing properties of the high-energy branch, a more detailed theoretical discussion can be found in our previous work [25].

**Figure 8:**
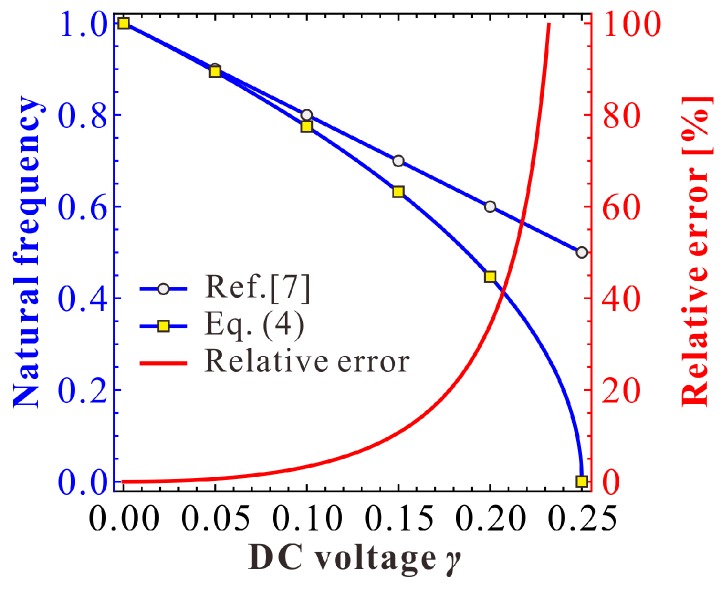
Nondimensional comparison depicting the natural frequency versus DC voltage.

Note that when the natural (angular) frequency of the resonator is away from one, the theoretical solution in Reference [7] gradually lose its prediction accuracy, especially on the natural frequency. Out of curiousity, we investigate the frequency shift phenomenon of the work in Reference [7] compared with the right one Equation (4). From Figure 8, we can find that the frequency shift phenomenon becomes obvious as DC voltage γ increases. This means that as the DC voltage increases, their theoretical solutions cannot be used for natural frequency and dynamic predictions. On the contrary, our present natural frequency is the same as in Equation (4). Thus, it is more convincing for vibration identification.

In this section, we theoretically give a vibration identification of the evolutionary pattern of the primary frequency response of this type of MEMS-folded comb drive resonator, and finally validate via RK-4 results. From a dynamic perspective, we can now explain the reason for the different primary frequency response in the system. Perhaps, these theoretical results can be more convincing after some validations via a specfic MEMS rsonator, which implies the main work in the following study.

### 4.2. Dimensional Analysis

**Figure 9:**
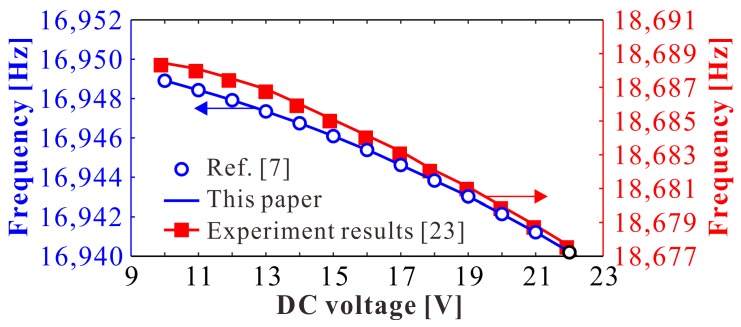
Natural frequency compared with experiment results in Reference [23].

Firstly, in order to verify the applicability of our theoretical results, we compare our natural frequency results with the experiment results in Reference [23], as shown in Figure 9. From this figure, we know that our present results are the same as theoretical predictions in Reference [7] when DC voltage is far away from pull-in voltage 613 V, which is consistent with the experimental results. Thus, our present study can be used for analysis.

**Figure 10:**
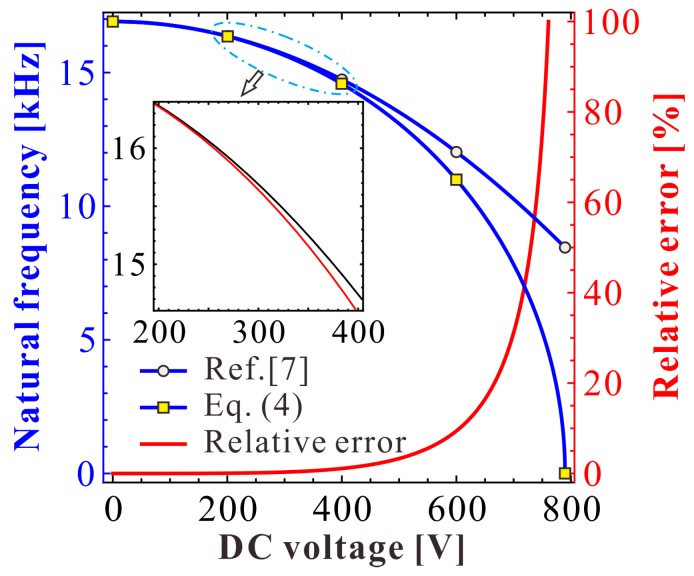
Dimensional comparison depicting the natural frequency versus DC voltage.

Next, a specific MEMS-folded comb drive resonator is considered for numerical analysis. The geometric and material parameters of a MEMS resonator are given in Table 1. Based on these parameters, we draw a comparison diagram depicting the natural frequency of the resonator versus DC voltage, as shown in Figure 10. Similar to Figure 8, DC voltage $V_{D}$ can indeed induce the frequency shift of the system. Besides, the theoretical prediction in Reference [7] is only available when $V_{D}$ is far away from the pull-in voltage.

**Figure 11:**
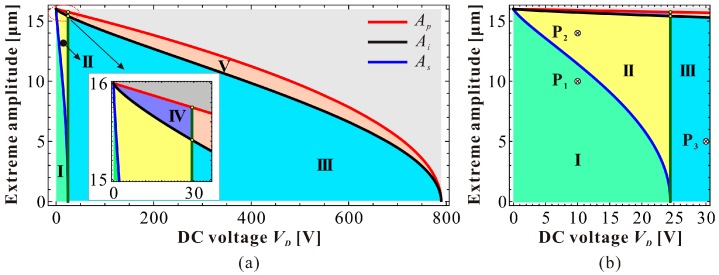
A dimensional zoning diagram depicting the extreme amplitude versus DC voltage. (**a**) Global view; (**b**) local view.

**Figure 12:**
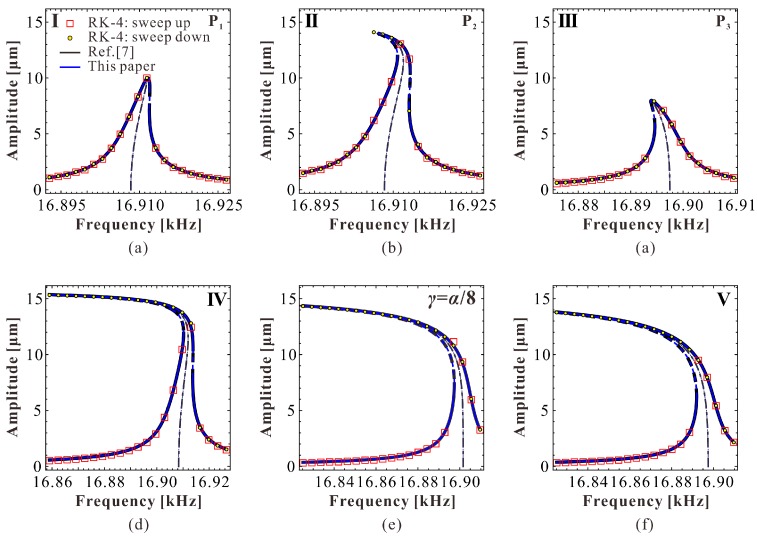
Frequency response curve with low energy corresponding to Figure 9. (**a**) $V_{D}=10\;V$, $V_{A}=60.46\;\mathrm{mV}$; (**b**) $V_{D}=10\;V$, $V_{A}=84.62\;\mathrm{mV}$; (**c**) $V_{D}=30\;V$, $V_{A}=16.11\;\mathrm{mV}$; (**d**) $V_{D}=10\;V$, $V_{A}=100\;\mathrm{mV}$; (**e**) $V_{D}=24.45\;V$, $V_{A}=40\;\mathrm{mV}$; (**f**) $V_{D}=30\;V$, $V_{A}=32\;\mathrm{mV}$.

**Figure 13:**
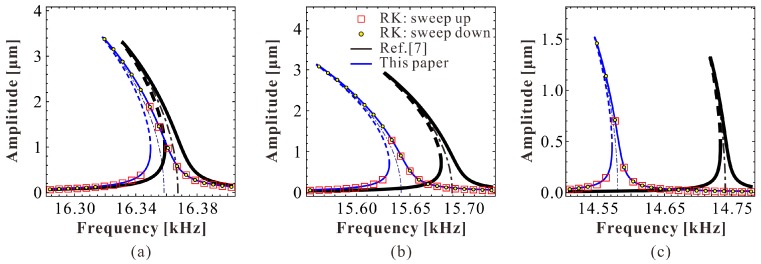
Frequency response curve with low energy as the DC voltage increases. (**a**) $V_{D}=200\;V$, $V_{A}=1\;\mathrm{mV}$; (**b**) $V_{D}=300\;V$, $V_{A}=0.6\;\mathrm{mV}$; (**c**) $V_{D}=400\;V$, $V_{A}=0.2\;\mathrm{mV}$.

Besides, we can depict a dimensional zoning diagram, as shown in Figure 11. P_1_, P_2_, and P_3_ correspond to extreme points with low energy. In the different regions of this figure, we can obtain various vibration patterns. The simulation results are shown in Figure 12. Under these parameters, when the DC voltage is small, both the theoretical results in Reference [7] and our present results coincide with numerical simulations. However, from Figure 13, one can notice that as the DC voltage increases, the theoretical results in Reference [7] gradually lose their prediction accuracy. Differently, our preset theoretical model keeps its accuracy compared with the numerical results.

## 5. Discussions and Conclusions

### 5.1. Discussions

**Figure 14:**
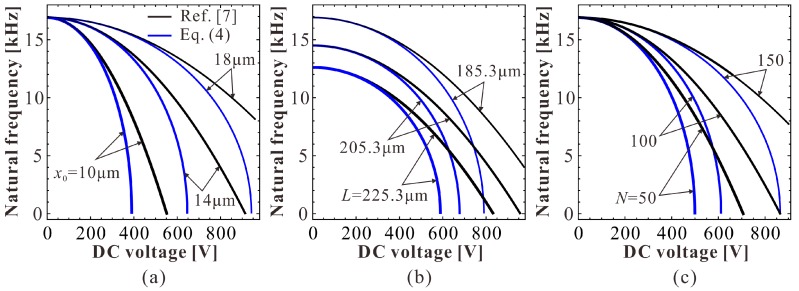
The effects of different physical parameters on the natural frequency of the resonator. (**a**) The effect of initial lateral spacing; (**b**) the effect of beam length; (**c**) the effect of total number of fingers.

According to Figure 12 and Figure 13, one can notice that the estimation of the natural frequency is a key factor during the dynamic prediction of the resonator. To discuss the frequency shift phenomenon in detail, comparison diagrams depicting the effects of different physical parameters on the natural frequency of the resonator are shown in Figure 14. Although the frequency shift can be affected by different physical parameters, there is one rule in these curves: the right natural frequency decreases from one to zero as the DC voltage increases. However, the theoretical estimation in Reference [7] cannot hold this variation property. For example, in Figure 14a, when $x_{0}$ is 10 μm, we can calculate from Equation (4) that the pull-in voltage is about 400 V. Then, the natural frequency should be zero. However, the natural frequency predicted by Reference [7] is about 8 kHz, which cannot effectively predict the pull-in instability of the system. Besides, as the DC voltage increases, the natural frequency in Reference [7] shows an obvious error compared with the right one. By contrast, our present results are the same as those in Equation (4). Thus, our present theoretical solution is more suitable for the vibration identification of this MEMS resonator.

The reason for this frequency shift phenomenon comes from the excitation assumption in Equation (6). Based on the MMS, the nondimensional natural (angular) frequency is assumed to be one. Once the natural (angular) frequency $\omega_{n}$ is away from one, the error of assumption in Equation (6) becomes obvious, inducing such an error as shown in Figure 14. Differently, our present result in Equation (12) derived from the backbone equation in Equation (11) is the same as the right one in Equation (4). Thus, our theoretical solution is available for all of the DC voltages. Based on our present solution, the primary frequency response can be totally predicted and verified.

For such a folded-MEMS comb drive resonator, the governing equation of motion in Equation (3) seems to be different from a traditional nonlinear dynamic equation with such quadratic or cubic terms [30]. The Hamilton system of Equation (3) includes heteroclinic orbits that can separate the phase space into two regions: a stable region and an unstable region. According to Figure 4, we can notice that the high-energy phase diagram is close to the heteroclinic orbits, indicating that the high-energy vibration has a certain basin of attraction. If this basin of attraction can be discussed in detail, then we can realize a large motion through using relatively small AC excitations. However, this vibration pattern is strictly dependent on the initial condition of the system; thus, it seems to be difficult to be triggered in practice. Actually, when both low and high-frequency branches intersect with each other, we may obtain high vibration energy via a frequency sweep-down operation. This seems to be much easier to realize. Perhaps, it may be applied in filtering or energy harvesting fields. However, much work still needs to be done to realize these designs.

### 5.2. Conclusions

To find convincing theoretical results for this type of electrically actuated comb drive resonator, one should focus on two key aspects. One is natural frequency. The other is dynamic estimation. Under these considerations, we investigated the vibration identification of the system. An improved solution procedure applied in our previous work was used here for primary resonance identification. For the first time, we found that high and low-energy frequency branches might exist in this comb drive system. A global zoning diagram depicting extreme vibration amplitude versus DC voltage was then drawn and discussed. Through comparison with previous results in the literature and numerical simulations, we verified the correctness of our theoretical results, and also pointed out the reason for their inaccurate prediction with large DC voltage. Based on these results, the primary resonance behavior of this type of folded-MEMS comb drive resonators can be identified in detail, which may be useful for some designs of comb drive resonators in the fields of MEMS sensing, actuating, and energy harvesting.

## Figures and Tables

**Table 1:** Geometric and material parameters of a MEMS resonator (refer to Reference [7]).

Parameters	Values
Mass, *m*	5.73 × 10^−11^
Quality factor, *Q*	5000
Beam length, *L* (μm)	185.3
Beam width, *W* (μm)	1.9
Initial overlap, *l* (μm)	20
Finger gap spacing, *d* (μm)	2
Structure thickness, *h* (μm)	2
Finger width, *w* (μm)	2
Initial lateral spacing, *x*_0_ (μm)	16
Young’s modulus, *E* (Gpa)	150
The dielectric constant, *ε*_0_ (F/m)	8.85 × 10^−12^
The total number of fingers *N*	60

## Appendix A

Elshurafa et al. [7] gave a theoretical solution of the average equation by the application of MMS, which can be expressed as:

(A1) $$\left\{ \begin{array}{c} \frac{dA}{dT_{1}}=-\mu A+2\gamma \delta \rho \sin\varphi \\ \frac{d\varphi }{dT_{1}}=\omega -1-\frac{3\alpha A^{2}}{8}+\frac{2\gamma }{{\left(1-A^{2}\right)}^{3/2}}+\frac{2\gamma \delta \rho }{A}\cos\varphi \end{array}\right.$$

where $\varphi =\sigma T_{1}-\beta$.

The primary frequency response can be given by:

(A2) $$\omega -1-\frac{3\alpha A^{2}}{8}+\frac{2\gamma }{{\left(1-A^{2}\right)}^{3/2}}\pm \sqrt{{\left(\frac{2\gamma \delta \rho }{A}\right)}^{2}-\mu^{2}}=0$$

The backbone curve can be expressed as

(A3) $$\omega_{0}=1+\frac{3\alpha A^{2}}{8}-\frac{2\gamma }{{\left(1-A^{2}\right)}^{3/2}}$$

when A=0, we can find the natural frequency $\omega_{0,0}$ as follows:

(A4) $$\omega_{0,0}=1-2\gamma$$

The stability of the system can be identified by Jacobi matrix:

(A5) $$J=\left[\begin{array}{cc} -\mu & 2\gamma \delta \rho \cos\varphi \\ -\frac{3\alpha A}{4}+\frac{6\gamma A}{{\left(1-A^{2}\right)}^{5/2}}-\frac{2\gamma \delta \rho }{A^{2}}\cos\varphi & -\frac{2\gamma \delta \rho }{A}\sin\varphi \end{array}\right]$$

Finally,

(A6) $$\lambda^{2}+R\lambda +S=0$$

where

(A7) $$R=2\mu ,\;S=\mu^{2}+\left[\omega -1+\left(\frac{2\gamma }{{\left(1-A^{2}\right)}^{3/2}}-\frac{3\alpha A^{2}}{8}\right)\right]\cdot \left[\omega -1+\left(\frac{2\gamma (1+2A^{2})}{{\left(1-A^{2}\right)}^{5/2}}-\frac{9\alpha A^{2}}{8}\right)\right]$$

when R and S are both positive, the vibration of the system is stable.
